# A road map to Translational Medicine in Qatar and a model for the world

**DOI:** 10.1186/1479-5876-10-177

**Published:** 2012-08-29

**Authors:** Francesco M Marincola, Javaid I Sheikh

**Affiliations:** 1 Office of the Dean, Weill Cornell Medical College in Qatar, Qatar Foundation, Education City, P.O. Box 24144, Doha, Qatar; 2 Clinical Center, National Institutes of Health, Bethesda, Maryland, 20892, USA

## Abstract

Translational Medicine (TM) in Qatar is part of a concerted effort of the Qatari medical and scientific leadership supported by a strong political will by Qatari authorities to deliver world-class health care to Qatari residents while participating in the worldwide quest to bridge the gap between bench-to-bedside-to-community. TM programs should embrace the Qatar National vision for research to become an international hub of excellence in research and development, based on intellectual merit, contributing to global knowledge and adhering to international standards, to innovate by translating new and original ideas into useful applications, to be inclusive at the national and international level, to build and maintain a competitive and diversified economy and ultimately improve the health and well-being of the Qatar’s population. Although this writing focuses on Qatar, we hope that the thoughts expressed here may be of broader use for the development of any TM program particularly in regions where an established academic community surrounded by a rich research infrastructure and/or a vibrant biotechnology enterprise is not already present.

## 

"*There are three golden rules for the effective treatment of any disease….*"

"*Unfortunately, we do not know any of them*"

## Introduction

Translational Medicine (TM) in Qatar is part of a concerted effort of the Qatari medical and scientific leadership supported by a strong political will by Qatari authorities to deliver world-class health care to Qatari residents while participating in the worldwide quest to bridge the gap between bench-to-bedside-to-community. Within a commitment from Qatar’s authorities to spend 2.8% of the government’s revenue in research and development, TM stands as part of the National Health Strategy 2011–2016 (NHS), to be conducted in accordance with the priorities established by NHS and coordinated by the recently formed Qatar Medical Research Council (QMRC). In addition, TM can and should provide creative solutions stemming from current basic and clinical science knowledge beyond those described by NHS. TM embraces the Qatar National vision to become an international hub of excellence in research and development, based on intellectual merit, contributing to global knowledge and adhering to international standards, to innovate by translating new and original ideas into useful applications, to be inclusive at the national and international level, to build and maintain a competitive and diversified economy and ultimately improve the health and well-being of the Qatar’s population. Although this writing focuses on Qatar, we hope that the thoughts expressed here may be of broader use for the development of any TM program particularly in regions where an established academic community surrounded by a rich research infrastructure and/or a vibrant biotechnology enterprise is not already present.

### Definition and Interpretation of Translational Medicine

a. A broad definition of TM leaves open and vague boundaries that allow a comprehensive understanding of its purposes but make difficult the clarification of its goals and application.

The Qatar National Research Fund (QNFR) recognizes three categories of research and development: basic research, applied research and experimental development; like basic research, *applied research* is “*original investigation but, contrary to basic research, it is directed primarily towards a specific practical aim or objective*”.^1^*Experimental development* is “*systematic work, drawing on existing knowledge which is directed to producing new materials, products or devices, to installing new processes, systems and services*”. Interestingly, in the Zerhouni Group, LCC (TZG) report consisting of a blue ribbon panel commissioned by the QF under the leadership of the former National Institutes of Health, USA Director Elias Zerhouni on “*Enhancing Qatar’s National Research Enterprise*”, a distinction is made between applied research and translational research: while *applied research* is “*designed to discover new knowledge that can help address the specific needs of Qatar and the region*”, *translational research “bridges the gap between bench-to-bedside-and-back to better understand disease epidemiology and specific characteristics in Qatar”*. An argument could be made that translational research or, more specifically, translational medicine sits astride applied research and experimental development.

We recently prepared an entry on translational medicine for the *Encyclopedia Britannica*[1] that encompasses previous definitions from our group [2-4]. We defined TM as “*research to improve health and longevity by determining the relevance to human disease of novel discoveries in the biological sciences. TM is a bidirectional concept, which encompasses bench-to-bedside factors that seek to increase the efficiency of therapeutic strategies tested in humans, and bedside-to-bench factors that provide feedback about the effects of treatment. Assessments are dependent on tools for the characterization of disease processes and the generation of novel hypotheses based on direct human observation*”. While this definition, as the ones mentioned in the previous paragraph, provides a comprehensive overview of the TM concept, in practical terms, it leaves open and vague boundaries that cloud the demarcation of targeted efforts and confuse the application of TM, particularly, when resources for distinct projects need to be prioritized. Therefore, a more stringent, “*working definition of TM*” might better guide TM activities particularly during the development stage.

b. A stringent definition of TM enlightens goals: what TM is and what it is not

The core of TM is the integration of novel technical developments relevant to biomedical discovery for the in depth understanding of biological processes directly related to human disease. In other words: how can the clinical sciences take advantage of the rapidly expanding knowledge and evolving technology to pursue health benefits? Can TM serve as a hub that integrates within one unit all the components relevant to solve a specific clinically relevant problem through *ad hoc* participation of all expertise from basic or applied research and from experimental development? This working definition implies that *the core of TM is the selection of a precisely defined, clinically relevant question that should drive the process*[5]. Thus, TM should not be confused with broader also clinically relevant initiatives that have, however, a less defined end point such as, for instance, the important activities initiated by research institutes or centers (e.g. the Qatar Biomedical Research Institute (QBRI), the Cardiovascular Research Institute, the Drug Discovery Center and the National Qatar Biobank), which play complementary but distinct roles. The scope of these initiatives is broader and overreaching while *TM should take advantage of close contact with human material prospectively obtained in clinical settings to answer carefully selected contemporary and salient clinical questions*.

### The goals of TM in Qatar

Qatar-specific TM goals should take into account the relatively early stage of the enterprise and the limited availability of human and technical resources. Thus, through a gradual process, a balance should be stricken between breath of scope and depth of output. Prove of concept initiatives should be carefully selected according to the following hierarchy:

c. Focus on the solution of health care problems prevalent in Qatar and the Middle East and North Africa (MENA) region to:

i. Address in the short-term immediate local and regional needs to improve the health and quality of life of the population

ii. Exploit scientific opportunities, while addressing local and regional needs, to contribute to the broader goal of understanding disease and improving treatment strategies of global impact

d. Tackle health care problems of worldwide impact though relevant to Qatar and the MENA region delivering cutting edge experimental care at par with top institutions across the world and to become a magnet for:

i. Qatar is currently seeking specialized health care services abroad

ii. The MENA community

iii. The worldwide community through the development of unique programs in specific areas of excellence

### Challenges and opportunities for TM in Qatar

We have extensively discussed in the past the obstacles generally faced by TM [6-8]. Here, we focus on topics pertinent to Qatar referring to general issues as relevant. The NHS outlines challenges and potential solutions to the improvement of health care delivery in Qatar; the goal is the provision of a “*comprehensive world-class healthcare system including: effective and affordable services, coverage of preventive and curative health care and high quality research directed at improving the effectiveness and quality of health care*”. Clearly, TM in Qatar should support and at the same time benefit from NHS initiatives; some best collimate with the purposes of TM and are highlighted below. As suggested by TZG, prioritization should follow a stepwise process:

e. Setting biomedical research priorities based on:

i. Relevance

ii. Risk-Reward ratio

iii. Return on investment

While setting priorities, it will be important to evaluate the potential of proposed projects to achieve the set goals within a reasonable time allowing at the same time flexibility for expansion from the original timeline upon achievement of encouraging milestones. Flexibility should be applied also to the reassessment of original plans according to promising and unexpected results. Nevertheless, using the potential scientific impact of a given proposal independent of outcome as a guiding principle for evaluation should safeguard from the selection of risky and open-ended initiatives with minimal likelihood of affecting the health of Qatar’s population in the first place and more broadly Qatar’s social and economic development.

A road map to the prioritization of salient health care problems in Qatar is provided by the NHS, here are outlined those mostly relevant to the establishment of a TM program:

f. Current Health Care needs to be prioritized:

i. Research Area Priorities in Qatar are outlined by the Rand Group Study [9], as summarized in the Qatar Foundation (QF)/QNFR website where salient funding programs are listed: Health & Life Sciences

In addition to these, other emerging priorities have been identified that include:

· Diabetes

· Obesity

· Cardiovascular diseases

· Women’s and children's health

· Genomic factors on health of individuals and population

· Cancer (breast, hematological, colon, and malignancies)

· Health and environmental pollution

· Infectious diseases

· Neurosciences (mental health, brain injury and epilepsy)

· Management of trauma

The TM program will need, at least at the onset, to adjust its focus according to the roadmap established by NHS. Here, we add specific comments illustrating how a TM program may provide original contributions for the achievement of these National goals. The chronic diseases outlined by the RAND Group Study are the major cause of mortality in Qatar accounting for approximately 50% of deaths and the prevalence is even higher among Qataris. The TM program should develop specialized experimental treatments for each of them by providing the clinical research infrastructure as later described in the strategy section. For instance, the National Cancer Strategy (NCS) [10] outlines an ambitious and comprehensive cancer treatment program spanning education, awareness, prevention, early detection and advanced tertiary care. Importantly, the NCS includes a research component “*by involving the diverse research community and the people of Qatar, new insights will emerge that can enhance the recommendations within the strategy*”. The TM program should support the research aspects of NCS by providing novel insights in the early diagnostic and staging of cancer; an example could be the participation to worldwide initiatives such as the colon cancer immunoscore project [11] sponsored by the *Society of the Immunotherapy of Cancer*[12], which is redefining cancer staging. The TM program may initiate in Qatar world class, cutting edge experimental therapeutic strategies, particularly early phase trials such the US National Cancer Institute Personalized Cancer Care/Drug Development Platform [13], testing of pathway inhibitors, check point and/or microenvironment modulators [14], immunotherapy [15] and cell therapy [16]. Few targeted proof of principle early phase clinical trials should be considered to attract Qataris to seek medical care in their own Country instead of going abroad for cancer care, while people from the Gulf region, and even further afield, will want to come to Qatar for cancer treatment. Several of these approaches are relatively simple to implement, on an outpatient basis and proven to be effective, vaccines against the development/recurrence of cervical [17,18] and lung cancer [19] could be tested. Similar approaches could be implemented for the study and the experimental treatment of diabetes, obesity [20] and childhood obesity [21]. Partnership with entities like the Sidra Hospital dedicated to Women and Child health will be important, particularly in areas like vaccination against human papilloma virus, or other childhood and adult vaccination programs for which even at the global level little is known about individual variability in response. Moreover, other interesting questions could be explored related specifically to the Qatari women such as the relationship between breast cancer prevalence/survival and vitamin D deficiency [22,23] in the context of suboptimal light exposure [24].

ii. It is important to outline some characteristics of the Qatari residents including Qatari nationals and expatriates, each with specific sets of health related issues. A recent census of the Qatar population can be accessed at [25].

According to the Qatar Statistics Authority, on September 30^th^ 2010, there were 1,624,235 Qatari residents, approximately 350,000 of whom being Qatari citizens. The remaining residents were expatriates mainly from South Asia and from non-oil-rich Arab states. There are sub-population-specific, health care related problems that need to be considered separately. Although this report is not intended to provide a comprehensive overview, some examples are deemed necessary. For instance, communicable diseases are more prevalent in the expatriate population and, consequently, the prevalence of cancer in the expatriates is also biased by the occurrence of pathogen-dependent neoplasia. This variation may affect the selection of screening and vaccination programs on one side and their success rate on the other. Trauma is a significant problem in Qatar, but while car accidents are responsible for death and disability across Qatari residents, work-related injuries are specific to the expatriate laborers. Women health also bears regional idiosyncrasies as outlined by the NHS. Perhaps, the most striking sub-population-specific peculiarity is consanguinity, which is specific to the Qataris as discussed later.

iii. TM should support and at the same time benefit from the “Disease Management Program” for chronic diseases

TM is a natural partner in the implementation of long-term disease management programs. Most in depth studies on chronic diseases such as diabetes, cardiovascular disease and cancer require long-term follow up that is best performed in the setting of a disease management program. TM can participate in a bilateral exchange by

a. Supporting long term treatment of chronic disease with:

· Novel therapies that can be applied when standard approaches fail

· Identification and testing of pre-clinical tools for the rapid screening of new therapeutic strategies

· Supporting patient stratification through the identification of prognostic, predictive and surrogate biomarkers

b. Benefit by collecting prospectively human material that could be studied longitudinally for the identification of determinants of:

· The natural history of disease

· Responsiveness to standard or experimental treatment

· Identification of novel prognostic, predictive, mechanistic and surrogate biomarkers

iv. How can TM be integrated with studies related to consanguinity –

The current rate of consanguinity among Qataris is 54%, the most common type occurring among first cousins (34.8%) making Qatar one of the highest prevalence areas in the world [26]. It is even likely that the rate of homozygosity is higher than the number of cases recognized to be directly related because marriage within close communities has been a long-established custom [27]. Consanguinity among first cousins corresponds to a 5% risk of having a child with severe or lethal medical conditions. The optimal solution to the problem is obviously prevention through pre-marital counseling. However, the current reality claims the need and offers the opportunity to study the genetics of recessive single-gene disorders. This, in turn, provides the opportunity for the study of gene-specific dysfunction through the development of a “functional mapping” program associated with a genetic counseling clinic; a scientific need of global impact. Moreover, the relationship between consanguinity and the severity of multi-genic complex diseases of adulthood [27] could be investigated in Qataris better than in most populations worldwide. For instance, is the high prevalence of type II diabetes in Qataris only related to socio-economical factors, or could endogamy and consanguinity play a role? Several lines of work have recently pointed at genetic determinants regulating glucose homeostasis [28] and affecting the susceptibility to acquire type II diabetes [29,30]. Genetics studies related to diabetes and obesity are currently being conducted at WCMC-Q as well as QBRI; a translational unit integrated with the current research activities could complement and facilitate these efforts. The same reasoning could be applied to cancer (i.e. breast cancer, [31]), cardiovascular disease [32] and other disorders [27]. Thus, a concerted effort involving all Qatari stake holders such as the Hamad Medical Corporation (HMC), the Shafallah Medical Genetics Center (SMGC), QBRI and WCMC-Q, facilitated by the TM program could tackle not only problems relevant to the Qatari population but address at the same time unsolved global genetic questions that have been poorly investigated [27] taking advantage of the uniqueness of the Qatari population: in this case these efforts could test the hypothesis of whether increasing genome wide heterozygosity leads to reduction in burden of common genetic diseases.

v. How can TM support efforts to build local capacity

The QF, with the establishment of Education City, has taken a leadership role among Countries in the Gulf Region and the MENA region in modeling the integration of higher education with research and development [4]. The TM program should actively participate in the education and training of clinical investigators at different levels bridging educational activities in the basic sciences provided by WCMC-Q with training opportunities at HMC, Women’s Hospital, Sidra, SMGC, other local hospitals and centers, and eventually the TM program (for training in TM research). Consequently the TM program should participate in building Qatar-based residency programs providing training in clinical and translational investigation. The latter will serve not only the need to build local capacity but also serve a global need. In particular, the high cost and delay of revenue due to prolonged medical training has limited the number of available clinical investigators worldwide [33,34]. TM in Qatar, supported by designated funds from the QF/QNFR should establish and manage competitive fellowships to attract young investigators worldwide and contribute to the solution of this global problem. This could be done by establishing an elite clinical investigation fellowship program with competitive salaries, research support and infrastructure similar to the clinical research fellowship programs established successfully at the National Institutes of Health (NIH) that rigorously complement clinical with basic research training. In addition, special incentives and programs should be established to facilitate a natural continuation from the TM fellowship into a clinical research faculty position in Qatari institutions including transitional appointments (senior fellowships) for those not quite ready to become independent investigators.

vi. How can TM be integrated with the healthcare data project and the E-health program

A current major challenge for the success of biomedical investigation is the integration of biological data with high quality and comprehensive clinical and epidemiological information. Thus, TM should support any attempt to integrate information and at the same time benefit from such efforts. The TM program should also provide open access to patient-specific information (respective of privacy rules) that may benefit the general purposes of the health care data project and in general medical practice in associated institutions.

vii. How TM can be integrated with the growing Public Health initiatives and an increased emphasis of primary care centers

TM can contribute to Public Health and Preventive Health care by identifying novel biological surrogate markers to monitor the effectiveness of preventive efforts particularly at the preclinical stage, which are currently not available for most chronic diseases. At the same time, a close relationship with primary care givers could start a bidirectional process for exchange of research material addressing prevalent conditions in the Qatar’s residents, while providing clinically useful information based on novel advances in biomedicine.

viii. Engaging basic scientists

The Federation of American Societies for Experimental Biology (FASEB) in association with the Howard Hughes Foundation, the Burroughs Wellcome Fund, the US Department of Veterans Affairs, the Doris Duke Charitable Foundation, and Merck Sharp & Dome Corp. organized a symposium on “*Engaging basic scientist in translational research: identifying opportunities, overcoming obstacles*” that resulted in a document providing guidelines and reporting on a survey inclusive or 2,000 investigators [35]. It should be recognized that basic science is the foundation of the biomedical research enterprise since it provides a logical interpretation of observations that facilitates solutions of medical problems through a systematic rather than empirical process. Yet, “*in spite of major advances in fundamental biology, there is widespread concern about the slow pace at which these discoveries are translated into sage and effective clinical interventions…Numerous initiatives to speed translation are under way, many of which have been aimed at providing clinical scientists with the knowledge and tools needs to translate research discoveries into improved patients care. Less attention, however, has been given to the contributions that basic scientists make to the process of translational research*” [35]. It was recognized that several obstacles face the basic scientist willing to participate in TM initiatives spanning the scientific, institutional, cultural and policy realm. In particular, it was recognized that while TM best fits a multi- and interdisciplinary *modus operandi*, the research environment encourages specialization and rewards individual achievement and hypothesis-driven, investigator-initiated research. We refer to the FASEB report for a comprehensive overview of this issue; however, it is pertinent to emphasize in this document the importance of engaging the basic science community in Qatar as anywhere else. This can be achieved by providing special rewards to supplement the classic academic process targeting those scientists wanting to significantly participate in TM initiatives, paralleling the model described later for the rewarding of clinical scientists directly involved with TM initiatives. In fact, nearly three-quarters of respondents to the FASEB survey indicated that their primary motivation to embark in TM efforts was to have in impact on human health [35] but their involvement was hampered by the rewarding system. Participation of basic scientists to TM and in general clinical research also benefits institutions; successful development of new drugs, devices and procedures attract patients who want to benefit from cutting edge research, and attract funding from other public and private sources at the international level.

There is also the practical reality that most often basic scientists work in locations afar from clinical scientists; this poses a physical barrier to vibrant interactions that bears bigger weight than generally recognized. For this reason it would be reasonable to create dedicated space on a per needed basis for basic scientists actively involved in TM research to encourage their part time presence within the TM community. In addition, special lecture series could be organized where related basic and clinical science topics should be sequentially presented and participation of all involved in the TM initiative should be encouraged or even enforced. Finally, as pioneered by the Moores Cancer Center, San Diego, CA, USA (as an example) space may be dedicated around TM facilities to host industrial activities and minimize geographic distance as a biotechnology incubator [36]. A similar model could be expanded to host few highly relevant basic science laboratories.

ix. Fostering public/private partnership

The development of a biotechnology corridor around the QF is encouraged and planned particularly through the creation of the Qatar Science & Technology Park (QSTP). QSTP has been quite successful in facilitating the engagement of the private sector with universities, as a base for multinational and national companies to establish programs in Qatar. However, currently, these initiatives are predominantly in the development phase and Qatar does not enjoy the nurturing environment of a rich biotechnology community surrounding some major academic centers elsewhere such as Stanford University, Harvard University, and University of California in San Diego, to use a few examples. This is, of course, not unique to Qatar as most academic centers both in developed and developing Countries are not necessarily adjoined by a large private enterprise endowed with affluent venture capital. Moreover, large pharmaceutical companies are distant geographically and not used to interact with Qatar and the MENA region. In particular, the relatively small population of Qatar (including Qatari and expatriates) may not lend to big Pharma interest in supporting large-scale clinical trials. How can these hurdles be overcome? The following may represent potential solutions:

· Provide incentive to industry limiting overhead financial burdens (indirect cost) that hamper public/private interactions in other Countries

· Decrease regulatory hurdles by surgically dissecting unnecessary from necessary regulations regarding issues such as protection of patients safety and privacy, conflict of interest regulations, intellectual property issues; this could be done by taking a leadership role rather than following; a world class bioethics research institute aimed at streamlining rather than enhancing regulation and working together with the TM program should be considered

· Encourage recruitment in high profile clinical trials from the surrounding MENA region offering free or reduced cost medical care (for experimental trials) and potentially travel support following the NIH model

· Focus on early phase clinical trials as proof of concept rather than late phase trials that may require larger patient populations

· Complement industry-sponsored trials with high quality correlative studies of high academic impact (rewarding the TM program) and valuable clinical impact for outcome interpretation (useful to sponsoring partner) providing matching funds and sharing potential intellectual property

· Provide areas of expertise in costly cutting edge technologies whose services could be offered in a collaborative form to small biotechnology enterprises that would not otherwise have access

· Provide financial and administrative infrastructure following the venture capital model pioneered by the Accelerator [37]. This model provides starting funds, management, laboratory space and financial expertise to competitive start-ups, therefore, facilitating the transition from the academic to the commercial world with the purpose of turning promising technologies into powerful business.

· Encourage academia/private partnership following a collaborative approach rather than a financial gain expectation. Often, the contribution of industry to the academic enterprise is disproportionally looked upon as potential funder rather than intellectual exchange. In reality, industry partners can contribute significantly to the research and development process and should be engaged from the onset.

g. Logistical challenges specific to Qatar

i. Small health research workforce (human resources)

ii. Institutional immaturity

There is a clear limitation in the current and the projected work force in Qatar for the next decade. The problem is compounded by the expected rapid growth in number of Qatari residents, particularly expatriates that will result in increased health care demand. Thus, as per the TZG report, the capacity of Qatar to “*conduct projects on a broad front is curtailed by its current small health research workforce and by institutional immaturity with… loose connections between research and the health care delivery system*”. In particular, it appears that, in the enthusiasm of building a world-class research infrastructure, several institutions with overlapping goals are sprouting. This is not necessarily a negative occurrence particularly if their activities can be effectively coordinated by the QMRC to avoid turf battles and overlapping projects but rather encourage complementarity of goals and resources. The NHS is taking several steps to enhance workforce planning, increase recruitment and retention and improve professional education *in loco*. At the same time, although facilities and infrastructure is rapidly being built there are still areas in significant need for technical support. As well articulated by the TZG report, “*too often, Countries that pursue a knowledge economy find themselves facing a disconnect between their aspiration and the reality of their existing human capacity and physical resources…. An unappreciated mismatch between what can be done, what should be done, and by who is the most common cause of failure…It will be important to strike a balance between breath of scientific representation and depth in areas deemed to be of national importance*”. We believe that it is also important to establish key collaborations and partnerships with institutions abroad to facilitate transfer of knowledge and technology following effectiveness principles well described by Thomas Friedman in “*the world is flat*” [38].

### Proposed strategy

#### Specificity and linearity of goals

The primary principle of the TM strategy will be to avoid overly ambitious and unfocused efforts as well discussed by Moore *et al*. [5] in regard to the planning of pilot studies. Goals and strategies should be clear and simple avoiding the addition of unnecessary complexity to already complex biological and clinical problems and, most importantly, sheltering lack of focus with preposterous intricacy *(“if we make it complicated enough nobody will have the courage to challenge it!”*). Individual projects should be defined, designed and pursued following the rigor applied to hypothesis-driven academic standards.

iii. Question-driven *vs* technology-driven strategy

This can be achieved by a concerted effort to identify the primary clinical needs of local and global impact to be tackled by the Qatar scientific and clinical community. In addition, projected clinical and scientific impact should be enforced as a guiding principle in project prioritization to encourage TM researchers to focus on scientific projects likely to link their research activities with substantial health improvements through the development of novel therapeutics or the creation of clinically useful commercial products

#### Prioritization

All initiatives should be assessed for impact and ease of execution as for the NHS recommendations; in particular:

iv. To assess the impact:

1. Importance of the issue being addressed by the initiative

2. The expected initiative’s time to impact lag

3. The urgency of the issue addressed

4. *Follow a sequential logic of projects (added, not in NHS)*

v. To assess the ease of execution:

1. The budgetary range

2. The level of skill needed

3. The initiative’s complexity

An example of integration between initial TM projects and the NHS prioritization strategy is one of the *“seven quick wins: update vaccination programs for adults”*. Target diseases are influenza, pneumococcal infection, tetanus, diphtheria and pertussis, human papilloma virus, and herpes zoster. Several of them also pertain to children or women health. Although such vaccination efforts are standard in their delivery, patient-specific outcome of vaccination is highly variable according to genetic makeup of patients and environmental exposure even among healthy individuals. For instance, at the trans-NIH Center for Human Immunology, one of us (FMM) was recently involved in a study following the response of 200 healthy volunteers belonging to a relatively homogenous population to seasonal and H1N1 influenza vaccination with an integrated genomics, functional genomics, deep phenotyping and outcome approach. This study highlighted huge variations in biological responses in healthy individuals that could be partly attributed to previous vaccine exposure and partly to genetic and biological characteristics (manuscript in preparation) Thus, a major opportunity arises to study the effect of vaccination among different population (Qatar ethnic diversity? due to a relatively homogenous core population: Qataris compared with the heterogeneity of expatriates), while supporting a prioritized NHS goal.

#### Short term focus – Hypothesis generating/testing clinical studies

**Figure 1:**
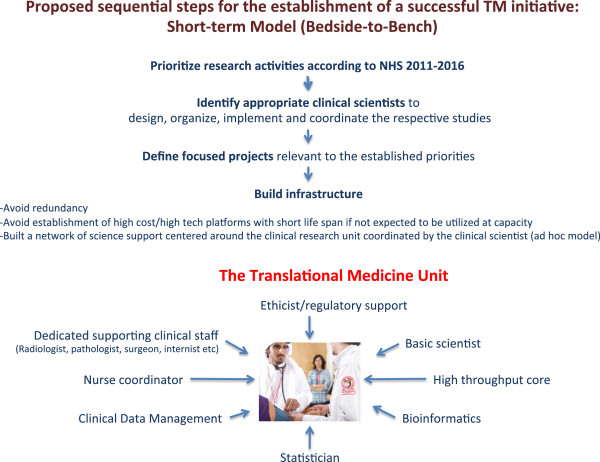
The short-term bedside-to-bench TM model.

At the onset, the TM program should focus on goals achievable in the short term following a bedside-to-bench strategy (Figure 1). The TM program could initiate correlative studies associated with standard of care treatment or off-the-shelf experimental therapeutic interventions to identify:

vi. Clinically relevant biomarkers suitable for commercial application

vii. Knowledge-generating studies to discover novel concepts elucidating disease patho-physiology that could foster subsequent investigations at basic, applied or developmental scientific level

This strategy combines the introduction in Qatar of emerging therapeutic concepts with provision of material for valuable scientific investigation. Critical to the success of this initial steps will be the creation of a National Clinical Trial Registry and develop mechanisms to facilitate awareness of clinical and research activities with the Qatari community, the MENA region and of global reach

#### Long term focus – Investigator-initiated clinical studies

**Figure 2:**
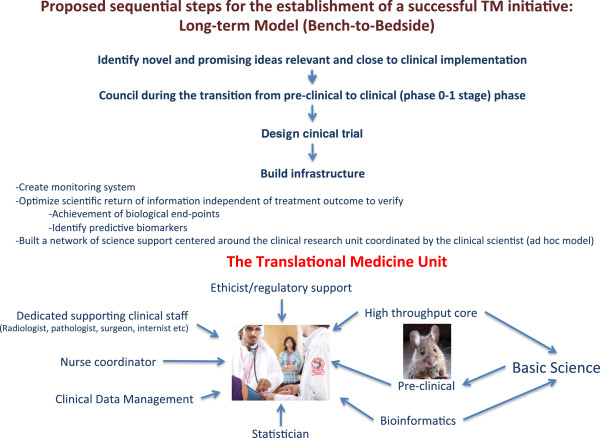
The integrated long-term bidirectional bench-to-bedside-to bench TM model.

Bench-to-bedside research is a long and costly process. Only a tiny minority of potential therapeutic candidates reach licensing for commercialization and clinical application after about a decade of testing and a cost in the range of billions [7,39]. Moreover, among licensed products, only a fraction is profitable. Therefore, it would not be wise to inaugurate the TM enterprise with product development as a primary, short-term goal. On the other hand, the TM program should be sensitive to and watchful for cutting edge, innovative ideas emerging from any of the Qatari academic institutions to support their testing and development in the clinical settings. TM investigators could help their basic science colleagues, starting from the envisioned final product and working backwards, to define the production pipeline of research steps required to support such product in the clinics. In addition, TM investigators should help the “go-no go” decision making process in late phase pre-clinical assessment before the cost in human resources and financial involvement escalates at the transition from laboratory investigation to clinical settings (Figure 2).

### Driving philosophy

As for many things in life, there are many ways to success or failure; most depend upon an optimized balance between a broad vision at the conception and sufficient attention to details during the implementation. We believe that important components to a successful TM initiative are: integrity, clarity, and transparency of goals and flexibility in execution. In our opinion, enterprises often fail because of lack of effective communication between the leadership, particularly of large institutions, and their constituency. Therefore, here we propose guidelines for the establishment of the TM program that do not span general concepts related to TM, as plenty has been written by us [1-3,6,8,40,41] and others [4,42-65]. Here, we rather focus on principles that are specific to the successful establishment of a TM program. We suggest that the following principles should serve as the foundation of a TM program in Qatar and that some may apply elsewhere.

#### Open and transparent decision making with a bottom up infrastructure

It is important for the leadership to maintain direct, continuous and effective communication with subordinates at all levels to optimize the balance between general goals and their implementation. The reporting relationships should not be excessively rigid sheltering and isolating the top from the bottom of the hierarchical structure. No matter how busy a leader might be, she/he should make a priority to communicate directly with and know his/her own work force. It does not take much to sample opinions directly: a good example is the “Undercover Boss” TV series by Stephen Lambert.

#### Goal-oriented, project-driven approach

The secret to success in TM is teamwork because the research endeavor requires different sets of skills directed toward a unified clinical question. However, most clinical research centers associated with academic institutions are currently following a “professional bureaucracy” model in which departments are segregated according to area of expertise, therefore, isolating specialties from each other. We suggest that TM should apply the “ad hocracy” model described by Mintzberg [66], which delineates a project-oriented structure where all elements necessary to the accomplishment of a given goal are included. For instance, this model is used for military special operations where effectiveness in achieving a goal is paramount. In the context of TM, we propose that the TM program should be constituted of separate units built around a clinical scientist, who functions both as principal investigator for TM unit-initiated projects and/or co-investigator facilitating projects initiated by other TM units or by other institutes but supported by and performed within his/her own unit. Dedicated scientific and clinical staff and resources should be recruited/assigned according to goal-specific needs and their utilization should be flexible. Staff and resources should be detailed to a TM unit on a permanent or temporary basis and according to a full or part time schedule according to need that can be periodically re-assessed. However, assignments should be person-specific for the duration of a project while rotations should be minimized. For instance, if a project does not require full time employment of a specialist (i.e. radiologist, pathologist, surgeon etc.) a part time appointment would be sufficient. However, the specialist should be selected according to specific interest and knowledge related to the project and should participate to all the intellectual activities and not only those pertaining to his/her own specialty. In other word, each co-investigator at any level should be an active component of all aspect of a given TM project. As a corollary, since it would be difficult to structure a unit around several goals, it is recommended that project selection should be carefully-evaluated according to impact of the question addressed, relevance independent of outcome and rigor of execution.

#### Team work, clinical relevance rewarding model

The current system of career advancement in science rewards primarily the individual; similarly publications list authors according to a rigid hierarchical structure assuming individual leadership roles and taking little account of team play. This creates a conflict between reward structure and expected results when multi-disciplinary projects are pursued, which are the essence of TM [6]. It is becoming apparent that large, cooperative and multi-disciplinary groups most often obtain significant contributions to the understanding of disease; the increasing number of publications in high impact journals demonstrates this with dozens of authors. Thus, the academic governance to which the TM program is reporting should implement a TM-specific reward structure. Professional recognition in the form of financial support and promotions should be granted according to impact of work rather hierarchical attribution in given projects or papers. It should be left to the principal investigators to evaluate which member of his/her team most effectively contributes to the TM unit goal(s). This may be challenging, because it introduces subjective judgment over the commonly applied objective parameters. Contrary to the co-investigators within each unit, each unit’s principal investigator should be judged according to the output of the unit independent of his/her hierarchical position in individual papers/projects. It is hoped that by holding each principal investigator responsible for his/her unit productivity rather than his/her own specific contribution, acknowledgement of contributions will be attributed proportionally to each individual’s participation rather than seniority and it will be in the principal investigator interest to provide accurate assessment of each member value to retain the most productive. In addition, projects should be evaluated not only according to scientific quality but according clinical relevance. Much has been discussed about parameters to achieve an objective evaluation of the clinical relevance of investigation [2,6,8,40,41,67]. However, no specific solution is currently available. We propose that clinical impact could be judged by the type of journals in which manuscripts are published (more geared toward clinical/translational investigations), the resulting effects in modifying clinical practice, and the output of potentially useful products as for instance suggested by granting of patents. In general, increased sensitivity to the clinical impact of ongoing investigations should be expected by scientific advisory councils judging TM activities: “*Tenure, promotion, and appointment committees should be challenged to evaluate the importance and impact of the individual contribution that a single faculty member makes in the context of a multi-investigator TM project*” [35].

### Required infrastructure

#### Substantiating the acquisition of costly equipment

TM does is not: *A crystal building with a genomics facility*. The rapid evolution of technology incessantly produces costly machines, requiring extensive training for their utilization, having a short life span before being outdated and requiring costly operational and maintenance support. Thus, their purchase is justifiable and cost effective only when the instrument can be utilized at full capacity and soon after acquisition. This is not always the case as several institutions fall to the temptation of obtaining “state of the art” equipment to show case without a clear vision of its utilization resulting often in instruments decanting in alleys, often unpacked. On the other hand TM cannot be insensitive to innovation. Thus, we suggest a mixed model to keep abreast with evolving technology: emerging technologies can be gradually tested in pilot projects through outsourcing even if the cost per sample is higher than projected given a full running in house system. This approach evaluates the usefulness and potential of a given technology with minimal capital expenditure, verifying at the same time the claimed validity. However, purchase of high technology costly equipment should be ultimately considered and encouraged when the novel technology is sound, long lasting and likely to be operated at close to capacity; this allows a cheaper overall operational cost per sample while enhancing the professional qualification of laboratory personnel.

#### The centralized (core facility) resources question

An obvious solution to underutilization is the optimization of capital investment into centralized/core units. However, frequently such units are underutilized [68] fail to provide the expected results and often are neglected by the potential beneficiaries due to close door policies, lack of transparency in priority selection, delays in service and obscurity in data interpretation. We believe that the core facility model in itself is not functional if not carefully crafted according to the needs and structure of an institution. Each unit, including centralized ones should be accountable according to academic standards, without privileged financial support rationalized as “service”. Centralized facilities should not be built as service providers but with associated research and development capabilities and be lead by a principal investigator responsible for their academic output using the same standards applied to other TM units. Financial support should be dependent upon substantiated academic output evaluated by the quality and relevance of publications for which the unit was responsible either primarily or in collaboration with others. In summary, a functional centralized facility should be assessed and rewarded according to evidence of collaborative work rather than been operated in a pay per fee service. Moreover, a functional centralized unit should be open providing access and teaching to TM investigators willing to learn sample processing, testing, and data analysis and interpretation and be accountable according to academic standards.

#### The special case of Bioinformatics and Computational Power

In the foreseeable future, while analytical platforms for genomics, proteomics, metabolomics etc. will continue to evolve, their output, standardized into computational units will converge into the pot of data analysis and integration; therefore, the true bottle neck of biomedical research will consist in limitations of transforming information into knowledge unless efforts will focus on keeping up with expansion of computational power and investment in programming tailored to scientific needs.

As the yield of high throughput technology logarithmically expands, corresponding needs arise in storage capacity, data transfer, organization and analysis to avoid the ever-growing bioinformatics bottleneck [62]. Moreover, software programs for interpretation of data have not caught up with the output of the novel technologies particularly when non-standard analytical approaches are required and when integration of information from different platform is involved. Interestingly, while several new technologies find rapidly a home in companies willing to provide as a service their implementation and, therefore, projects can often outsourced at a reasonable cost, it is unthinkable to relinquish data handling and interpretation to outside sources. This model has failed consistently since only the investigators in close contact with the analysts can lead the logical steps with the appropriate focus, specificity and accuracy. Thus, a TM unit should include or be closely associated with a TM bioinformatics support unit that includes:

· Information technology support

· Programming capability

· Mathematical modeling

· Computational biology/statistics expertise

The TM bioinformatics unit should be primarily responsible for the development of the infrastructure necessary for handling and processing large data sets, guiding scientists and their staff (research fellows, biologists) in study design with regard to bio-statistical power, analysis performance and presentation. However, we suggest that the TM bioinformatics unit should not run standard data analysis but rather guide scientists and their staff in performing their own analyses while the bioinformaticians/statisticians should devote their time in creation of new programs for high level, non standard solution to ever growing computational needs. We argue that, in the context of standard bio-statistical analyses, the computational biologists’ and statisticians’ time is better used in mentoring research fellows rather than performing analyses. This strategy will 1) protect the biostatistician time that can be dedicated to more creative activities; 2) provide a valuable teaching experience for scientists and fellows in an emerging scientific arena; 3) assure that the analytical process follows a logic process direct through the contribution of the scientist(s) who initiated the study. Finally, the development of novel analytical strategies should be the primary academic goal of the TM bioinformatics unit and should be rewarded with primary authorship in manuscripts and/or copyright privileges.

#### Establish targeted sponsorship from QF/QNRF or other sources

TM should receive dedicated and stable financial support from the sponsoring organization (supposedly the QF/QNFR) for operative costs and equipment. Support should be proportional to the breath of the programs agreed upon by the supporting parties and the TM leadership. Moreover, financial support should be dependent and adjusted according to the successful achievement of clearly established short and long-term targets and milestones. Some European institutions are implementing a formulaic target-dependent sponsorization system whereby operational costs are supplemented based on metrics such as individual publications and their calculated impact, development of patents and licenses, establishment of biotechnology enterprises, alternative support metrics (such as industry support), provision of matching funds associated with grants or donations from a third party, etc. Finally, TM investigators should be able to compete not only in general granting processes but also funding mechanisms through an independent sub research mechanism dedicated to TM. Currently, the QNFR defines specific sub research areas related to health among which Clinical Medicine and Basic Medicine take the lion’s part. We suggest that a sub research domain should be created dedicated to Translational and Applied research with clear demarcation of the goals and purposes of the discipline to encourage applications for high quality local and international projects.

#### Establish a Chief Scientific Officer (CSO) position (distinct from the Scientific Director)

Key to the success of the TM program is the hiring of a CSO who will work closely with the Clinical and/or the Scientific Director(s) and be responsible for managing the research and technological operations. In particular, the CSO will be responsible for supervising and coordinating the development of new processes, technologies or products particularly when they require significant capital investment and/or interactions among distinct TM units or other institutions. Institutional leadership is often focused on the “big picture” building harmony between the vision of a given institution, the local relevance, its worldwide impact, and the surrounding intellectual and political reality. While these skills are fundamental for the survival of the institution, they rarely come in association with detailed technical knowledge of the processes required to implement the vision. This particularly applies to biotechnology and, consequently TM, as methods and tools evolve rapidly and understanding of their potential value requires continued hands on evaluation. Thus, too often the implementation of the vision is scattered without coordination among lower ranking investigators following a principle reminiscent of the “trickle-down economy”: the leader provides the financial support and the scientists, as a community, will produce something that will approximate the envisioned product. This strategy, however, dos not apply to TM initiatives, which are often complex, thirsty for ever developing and costly technologies and multi-disciplinary in character. Thus, a manager capable of co-coordinating efforts from the bedside-to-the-bench and *vice versa*, with in depth expertise and hands on knowledge of all relevant components of the process from tissue procurement, to handling, processing, testing and analysis is necessary to avoid misuse of resources. Take this example (just one of many): company X sells a new technology; instrument cost $ 1,000,000; Dr. Y wants the TM program to buy it as part of the general operative costs (rather than his/her own budget) with the rationale that the new technology will eventually benefit other investigators, while his/her own laboratory will not utilize the instrument at full capacity. How will the decision making process be performed? While the Scientific Director may very well consider the relevance and impact of the proposed purchase in the context of the institutional vision, who could knowledgably dissect: what standard operative procedures for sample collection, storage and preparation suitable for the new technology will be required; how do they relate to current collection procedures and available resources, what are the projected operational and maintenance costs associated with the instrument, what is the projected proportional utilization related to the capacity and life-span of the technology, what alternative technologies should be considered, what is the proportional value of outsourcing rather than purchasing, what level of training of personnel will be necessary for the technology to be implemented efficiently, what kind of support for maintenance will be available from the company when the instrument is purchased, how will the output be handled, what data storage and processing tools will be required? It could be argued that the investigator or the director of a centralized unit relevant to the purchase could deal with such questions; in reality, however, an unbiased individual within the leadership, who can link directly scientific requests with the overall goals of the institution, can only make this decision-making process objectively. In summary, the CSO will flank the Clinical and/or the Scientific Director (s) of the TM program by providing internal expertise to fulfill with “hands on knowledge” the mission by bridging distinct disciplines coordinate development and efficiently execute the projected goals.

### Proposed time line and prospective metrics to measure success

It is time for TM efforts to yield results; a large number of TM institutes have been and are being created worldwide in the last decade. Most have failed and will fail in yielding the valued end product: a change in the standard of care. It is our personal opinion that the failures are largely due to insufficient attention to details, lack of focus, and unclear prospectively defined milestones and targets in the short and in the long-term. Thus, we believe that for the successful development of TM in Qatar (and elsewhere) these guidelines should be prospectively defined and followed to judge success (here general targets are listed while details for the evaluation are discussed in the subsequent section, milestones can only be negotiated later in the process of establishing the TM program):

a. Clear improvement in health care at the regional and global level

b. Academic success measured according to scientific contributions in the clinical research arena

c. Economic impact

d. Development of a critical mass of scientific/biotechnology enterprise around the TM program including a biotechnology corridor (incubator) attractive to world lass investigators

To implement a balanced investment portfolio, the metrics to success should be adjusted according to 2 timelines:

· Short-term goals

TM initiatives should at the onset focus on short term, realistically attainable results. We suggest that focused clinical questions answerable through analysis of already available or easily obtainable clinical material should drive the selection of the first generation projects; this bedside-to-bench approach could focus on the identification of useful biomarkers for patient stratification, to support the Public Health initiatives and for provision of a road map for future investigations. They should at the same time generate information and knowledge of significant academic impact.

· Long-term goals

Second/advanced generation studies should focus on impact and not only on feasibility: these studies could follow a bidirectional approach (bedside-do-bench or bench-to bedside and include 1) the design of complex prospective clinical trials based on experimental and/or standard therapies as single agents or in combination with the purpose of understanding mechanisms of action, identification of relevant biomarkers and definition of better therapeutic strategies [69-71] or; 2) investigator-initiated clinical studies to test novel therapeutic or diagnostic concepts.

### Evaluation process

#### Performance Metrics should be prospective

Targets and milestones should be clearly defined from the beginning and in line with the vision established in accordance with the NHS, QF and all other involved stakeholders. Regular though not overburdening reporting should be implemented on an annual basis while a periodic internationally based peer review process should be established. We recommend a “*retrospective focus*” in the review process looking at past achievement with minimal bearing on future initiatives. We believe that intellectual freedom and ability to “think out of the box” is a requirement for a creative approach to TM and the TM scientists should be allowed complete intellectual freedom in study design being judged only on the impact and relevance of their end product. However, as defined in the subsequent section, the peer review process could suggest new and unexplored concepts that could integrate Qatari efforts with worldwide interests.

#### Specific performance metrics and programs evaluation

viii. Operational objectives and milestones

1. Establishment of Faculty Awards to develop research laboratories in Qatar relevant to TM

2. Recruitment of Clinical/Translational Investigators

3. Implementation and coordination of Bioinformatics activities

4. Implementation of proof of principle pilot projects

5. Implementation of summer fellowships related to TM

6. Organization of international conferences

7. Establishment and coordination of core facilities

8. Establishment of administrative support

9. Definition and implementation of core research projects

ix. Scientific achievements should be judged according to the definition of clear indicators that are specific, measurable, achievable, relevant and timely:

1. Publication record in peer-reviewed journals

2. Citations indices such as for instance the H Index

3. Research collaborations at national and international level

4. Research Awards

x. Program evaluation should include:

1. Annual reporting to the sponsoring institutions describing the scientific and administrative operations of the program as well as planned activities for the following year(s). In addition, a budget of review and approval for each program should be presented

2. Periodic (biannual) evaluation by an international Scientific Advisory Board comprising internationally distinguished scientists. The Board should:

a. Be aimed not only at evaluating the current results but allowed to provide novel ideas and suggestions relevant to global health

b. Provide an opportunity to showcase the best achievements of TM in Qatar and “spread the word” by informing leaders in the field

## Summary and conclusions

The need to conduct biomedical research with relevance to and impact on human suffering is summarized by the TM concept; however, success will depend ultimately in the effective implementation into a functional reality. The development of a coordinated TM program, understanding the theoretical and practical hurdles facing such implementation and embracing possible solution with the necessary rigor and attention to details will ultimately determine the achievement of quality results. Here we propose basic ideas that may help in the conceptualization of TM programs starting from its broad scope to the focused implementation of individual goals. Although the focus is on Qatar, we hope that this exercise will be of use for the implementation of other programs elsewhere in the world particularly in areas where a rich surrounding infrastructure supported by an established academic community and/or a vibrant biotechnology enterprise is not already present.

## Endnotes

^a^In this manuscript unless otherwise specified, we will quote in *italic* passages from two documents: the National Health Strategy 2011–2016 (NHS) or the Zerhouni Group LCC (TZN) study on “Enhancing Qatar’s National Research Enterprise”. For more details please refer to the corresponding authors.

## Abbreviations

CSO, Chief Scientific Officer; FASEB, Federation of American Societies for Experimental Biology; HMC, Hamad Medical Corporation; MENA, Middle East and North Africa; NCS, National Cancer Strategy; NHS, National Health Strategy 2011–2016; NIH, National Institutes of Health; QBRI, Qatar Biomedical Research Institute; QF, Qatar Foundation, QMRC, Qatar Medical Research Council; QNRF, Qatar National Research Fund; QSTP, Qatar Science & Technology Park; SMGC, Shafallah Medical Genetics Center; TM, Translational Medicine; TZG, the Zerhouni Group, LCC (referred here as the report on Qatar’s National Research Enterprise); WCMC-NY, Weill-Cornell Medical College in New Your; WCMC-Q, Weill-Cornell Medical College in Qatar.

## Competing interest

Both authors declare that they have no competing interests

## Authors’ contributions

Both FMM and JIS contributed equally to the preparation of the manuscript by evaluating the current literature, analyzing relevant documents available through the Qatar Foundation recent activities and preparing the manuscript. Both authors read and approved the final manuscript.
